# Good to the last drop: The emergence of coffee ringspot virus

**DOI:** 10.1371/journal.ppat.1007462

**Published:** 2019-01-10

**Authors:** Michael Goodin, Antonia Dos Reis Figueira

**Affiliations:** 1 Department of Plant Pathology, University of Kentucky, Lexington, Kentucky, United States of America; 2 Universidade Federal de Lavras, Departamento de Fitopatologia, Caixa, CEP, Lavras, Minas Gerais, Brasil; University of Kentucky, UNITED STATES

## Introduction

**Fig 1:**
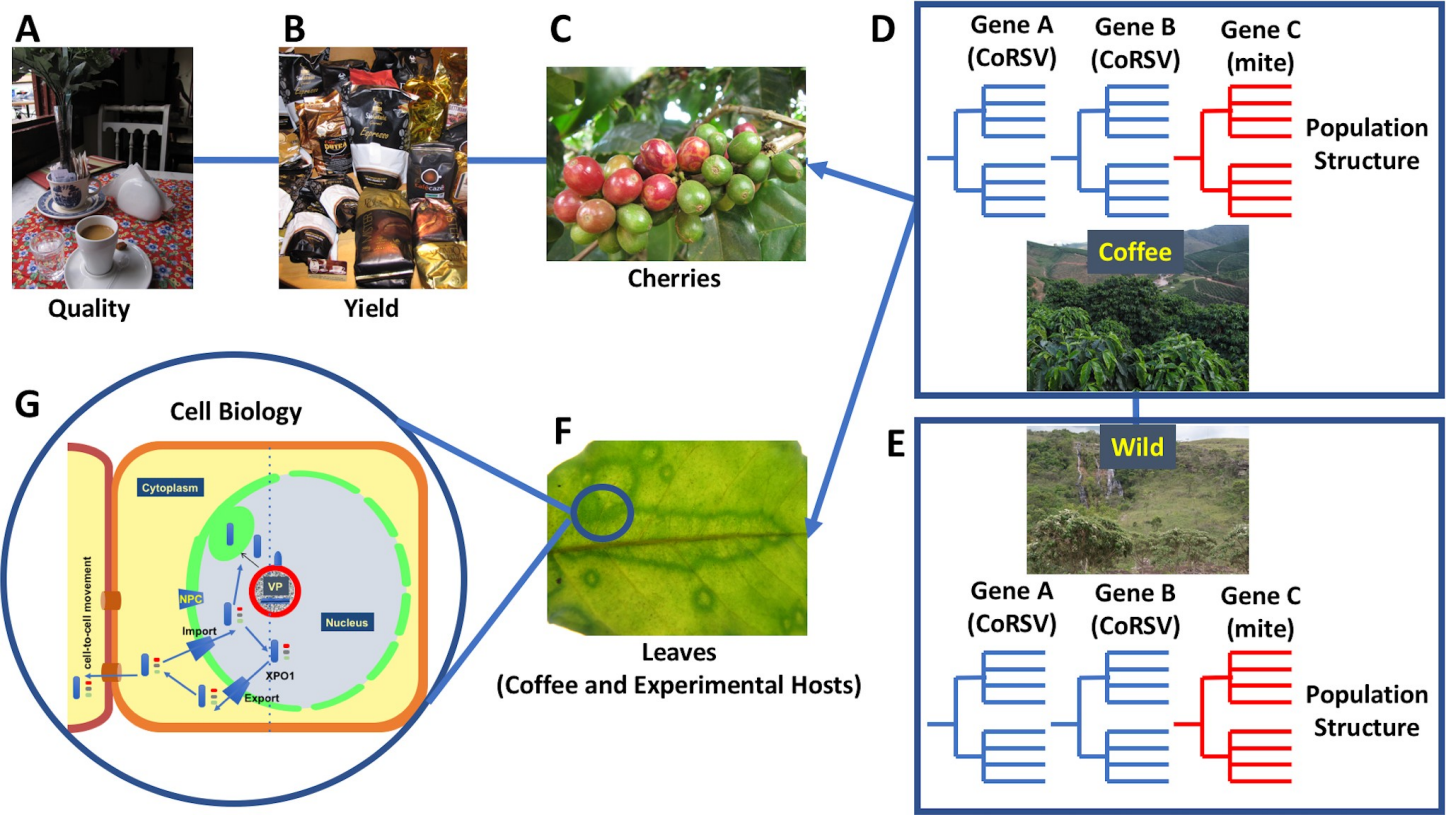
Diverse array of research projects related to understanding the effect of CoRSV on coffee. (A) Whether enjoyed alone or in the company of others, coffee is an integral component of daily life in all countries around the world. Only one small-scale study has been done to determine the effect of CoRSV on coffee quality [34]. (B) The effect of CoRSV on the yield of coffee plants has not been examined. (C) No formal investigations have been made to determine how CoRSV influences the development of coffee cherries. (D) Only one study has investigated the population structure of CoRSV [8, 35]. It remains to be determined if phylogenetic trees derived from different CoRSV genes or from viral RNA isolated from plants or mites are congruent. Furthermore, evidence for recombination or reassortment of CoRSV genomes has not been investigated in detail. (E) The reservoir of CoRSV in wild species, particularly in the Cerrado of Brazil, has not been investigated. It is unknown if the population structure of CoRSV in the wild is similar to that in coffee plants. (F) The molecular basis for temperature dependent susceptibility to systemic infections has not been determined [23]. (G) The cell biology of CoRSV beyond generation of protein interaction and localization maps is poorly characterized, particularly with respect to identification of host factors required for replication and cell-to-cell movement, viroplasm formation, and nucleocytoplasmic trafficking of CoRSV nucleocapsids and proteins [23]. CoRSV, coffee ringspot virus; NPC, nuclear pore complex; VP, viroplasm; XPO1, Exportin 1.

Two and a half billion times per day a human hand reaches for a fresh cup of coffee. Although arguably dispensable for life per se, with an industry value of US$174 billion, coffee provides the lifeblood that sustains economies of producing countries located in the “coffee belt” situated between the Tropics of Cancer and Capricorn. As a “solvent” in which many human interactions take place, coffee is witness to the broad spectrum of human activities from the mundane to the pleasurable and personal. However, in opposition to its economic, cultural, and physiological importance, diseases such as coffee rust (caused by the fungus *Hemileia vastatrix*) dictate activity on stock markets with their periodic epidemics, which in turn affects the migration patterns of displaced farm workers [1]. Other diseases, such as those caused by coffee ringspot virus (CoRSV), currently fly mostly under the radar of many integrated pest management systems. The unique biology of this and related viruses offers exciting research opportunities ranging from cell biology, plant pathology and physiology, conservation ecology, to climate change-related epidemiology. This review highlights important aspects of CoRSV, including its unique features, and examines the potential role of climate change in its emergence (Fig 1).

## CoRSV: Its genome organization and occurrence

**Fig 2:**
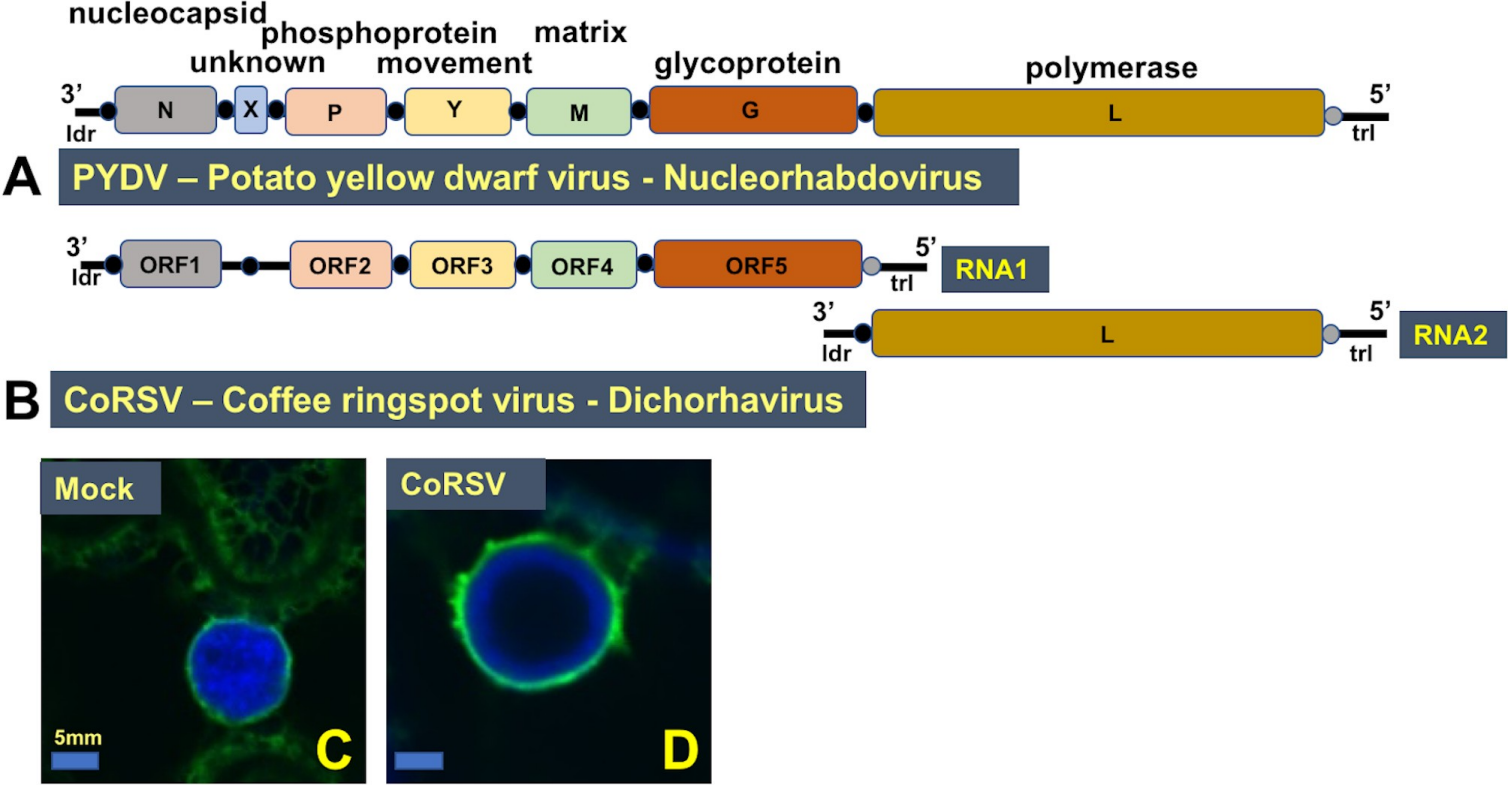
Genome organization of plant-adpated negative-strand RNA viruses. Colors represent proteins of similar function. Each open reading-frame is flanked by a conserved gene junction (black circle). Gene junctions lacking transcription start sites are located between the polymerase gene and trailer RNA regions. Note: “Y” is a standardized notation used here to denote movement proteins. (A) PYDV. Type species of the genus *Nucleorhabdovirus*. (B) CoRSV. Genus *Dichorhavirus*, which have bipartite genomes. Beneath the genome maps are overlays of single-plane confocal micrographs of virus-infected nuclei in leaf epidermal cells of transgenic *N*. *benthamiana* plants expressing GFP targeted to endomembranes (green). Nuclei were stained with DAPI to visualize chromatin (blue). (C) Mock. shown on left of virus-infected nuclei. Nuclei in virus-free cells have a clearly defined nuclear envelopes, with nuclei being approximately 10 μm at midsection. Scale is 5 μm. (D) CoRSV. Nuclei in these cells contain large viroplasms that exclude DAPI staining. However, the nuclear envelope is largely intact. Note that virus-infected nuclei are larger than virus-free nuclei. CoRSV, coffee ringspot virus; DAPI, 4′,6-diamidino-2-phenylindole; GFP, green fluorescent protein; ldr, leader; ORF, open reading frame; PYDV, potato yellow dwarf virus; trl, trailer.

In contrast to the members of the *Nucleorhabdovirus* genus (Mononegavirales) to which they are most closely related [2], members of the *Dichorhavirus* genus have bipartite genomes, although their coding capacity is about the same as that of the plant-adapted rhabdoviruses (approzimately 14 kb) [3]. All dichorhaviruses are transmitted by species of false spider mite, *Brevipalpus* spp., with orchid fleck virus (OFV) being the type species [3, 4](Fig 2).

CoRSV shares a pattern of emergence observed with numerous other plant viruses in being described decades ago and then rising into prominence as cultural and environmental conditions conducive to range expansion of their vectors are met with increasing frequency [5, 6]. First documented in 1938 [7], CoRSV is now established over the majority of coffee growing regions in Brazil [8]. A survey of some of these regions found CoRSV on 100% (n = 45) of the farms visited. Although the incidence of CoRSV varied greatly from farm to farm, the ease by which the virus could be found at every location was a significant and surprising finding.

Phylogenetic analyses of the *N* gene in 45 CoRSV samples were conducted in order to provide insight into the population structure of this virus within and between farms [8]. These studies revealed a strong geospatial relationship among isolates, given that the genetic distance between any two isolates was a function of the distance between collections sites. These data support the hypothesis that the spread of CoRSV is constrained by expansion of populations of Brevipalpus, which exist as haploidized females due to commensal interaction with *Cardinium* spp. This fascinating biology makes this arthropod an exciting subject for phylogeography studies in its own right beyond its impact as an agricultural pest [9].

An important and much under-investigated area in CoRSV research is identification of its wild reservoir hosts. Much of the coffee growing regions of Brazil are surrounded by wild and semiwild sections of the Cerrado [10–13], a tropical savannah in which the flora have not been extensively indexed for viruses, despite the ease with which plants in this region can be found with virus-like symptoms (Fig 1). Given the range of experimental hosts of CoRSV, it stands to reason that plants in the Cerrado may serve as reservoir species for CoRSV [8, 14]. Current virus-discovery-by-sequencing methods [15–17] are ideally suited to mapping the virus population structure of the Cerrado, which is the second largest savannah ecosystem in the world, with exceedingly rich biodiversity—much of which is undescribed—and under threat from human activity [18].

### Is there evidence for reassortment and recombination of the CoRSV genome?

That the genome of CoRSV, which in all other aspects resembles that of rhabdoviruses with monopartite genomes, evolved a bipartite organization begs the question whether this occurred due to the genetic constraints imposed by its unique vector. A bipartite genome may allow for increased opportunities for reassortment, if not recombination. Further, phylogenetic investigations based on whole genome and genes other than the nucleocapsid are required to provide a detailed phylogeography of this virus and its vector [18–21]. Given the low genetic diversity observed and the strong geospatial relationship between isolates, it might be expected that the phylogenetic trees derived from different CoRSV genes would be congruent (Fig 1). Although reassortment of CoRSV genomic segments has not been determined, it is clear that this mechanism for exchange of genetic material is possible in dichorhaviruses, based on investigations with OFV [22].

### What is the molecular basis for temperature-dependent susceptibility to CoRSV?

Some plant hosts, *Chenopodium quinoa* and *Nicotiana benthamiana*, for example, exhibit a dramatic temperature-dependent susceptibility to CoRSV [23]. In experiments conducted with both species, plants must be incubated at 28°C for at least five days in order for CoRSV to establish systemic infections. That this phenomenon occurs in two genetically dissimilar plant species suggests that the temperature-dependence affects some virus-specific process. The 2 to 4°C increase in ambient temperatures projected by climate change predictions may severely impact the occurrence of this virus in reservoir species, which in turn may impact the severity and frequency of CoRSV in coffee production areas.

### New resources required to support CoRSV research

Infection of plant cells by dichorhaviruses, or the related nucleorhabdoviruses, results in dramatic modification of nuclei without triggering programmed cell death or rendering nuclei nonfunctional [23](Fig 2). A number of new resources are required to gain insight into the molecular events that underlie CoRSV–plant interactions. Most important among these is the ability to recover infectious virus entirely from complementary DNA (cDNA) clones, as has been accomplished for sonchus yellow net virus (SYNV), after decades of sustained efforts [24]. Improved cloning strategies are effective for facile construction of infectious clones of rhabdoviruses and viruses with long RNA genome such as potyviruses. Such approaches should be applicable to a broad diversity of virus types [25], including dichorhaviruses.

Although recombinant systems will facilitate mechanistic studies of functional domains in viral RNAs and protein, the study of plant nucleotrophic viruses will be advanced substantially with a more detailed characterization of plant cell nuclei [26]. With the dramatic remodelling of nuclei by plant-adapted negative-strand RNA viruses, an essential area that is presently understudied is the characterization of protein dynamics in response to virus infection. Characterization of the portion of the plant proteome that associates with, or is resident in, nuclei lags behind that of yeast and mammalian systems. However, in a screen for novel nuclear proteins, the Goodin lab has identified several candidate proteins that will provide vital markers for mapping the response of nuclei to infection by CoRSV and related viruses [27]. As each of the plant-adapted negative strand RNA viruses examined to date has a unique protein interaction and localization map [23], it is anticipated that it will require investigation of a spectrum of viruses to identify common and unique features of plant–virus interactions. Several nucleorhabdoviruses and CoRSV can be studied in the common host *N*. *benthamiana*, for which genomics, biochemistry, and cell biology resources are rapidly expanding [28]. CoRSV has the additional advantage given that it has been shown to replicate in the model plant *Arabidopsis thaliana* [29].

### Is CoRSV trying to tell us something?

For the foreseeable future, coffee will remain “the best part of waking up” and all that follows thereafter in the spectrum of human social activities. That said, climate change forecasts promise little more than hardships for coffee producers [30], due to expansion of the range and prevalence of warm temperature pests such as the coffee borer [31], and *Brevipalpus* mites, the vector of CoRSV, while having a negative impact on beneficial pollinators critical for high yields [32]. Warmer average temperatures mean that diseases such as coffee rust will be able to reach higher elevations that are traditionally relatively free of this disease, a fact certainly vital to organic coffee producers.

Dire predictions aside, it is safe to say that no one associated with any part of the coffee industry, from grower to barista, is ready to capitulate to forecasts relating to climate change. To the contrary, the “Third Wave” of coffee is delivering a rapidly expanding diversity of specialty coffees to the world, and coffee-producing areas are taking on a regional and/or terroir aspect long associated with wine, as consumers increasingly relish the nuanced variation that microclimates have on coffee quality, aromas, and flavor, in addition to increasing interest in the geography and personal well-being of coffee growers per se. The dire outcomes of climate change on coffee production specifically, and agriculture in general, may not manifest themselves if governments of the world exercise the necessary interventions [33]. Otherwise, that “last drop” of coffee may be realized.
